# Genome-wide analysis reveals the association between alternative splicing and DNA methylation across human solid tumors

**DOI:** 10.1186/s12920-019-0654-9

**Published:** 2020-01-06

**Authors:** Xiaohui Sun, Yiping Tian, Jianming Wang, Zeyuan Sun, Yimin Zhu

**Affiliations:** 1 Department of Epidemiology & Biostatistics, School of Public Health, Zhejiang University, Hangzhou, 310058 Zhejiang China; 2 Department of Pathology, Zhejiang Cancer Hospital, Hangzhou, 310022 China; 3 Department of Radiation Oncology, Rutgers Cancer Institute of New Jersey, Robert Wood Johnson Medical School, Rutgers, the State University of New Jersey, New Brunswick, 08901 USA; 4 Department of Health Related Social and Behavioral Science, West China School of Public Health, Sichuan University, Chengdu, 610041 China

**Keywords:** Alternative splicing, DNA methylation, Exon boundaries, Cancers, Association

## Abstract

**Background:**

Dysregulation of alternative splicing (AS) is a critical signature of cancer. However, the regulatory mechanisms of cancer-specific AS events, especially the impact of DNA methylation, are poorly understood.

**Methods:**

By using The Cancer Genome Atlas (TCGA) SpliceSeq and TCGA data for ten solid tumor types, association analysis was performed to characterize the potential link between cancer-specific AS and DNA methylation. Functional and pathway enrichment analyses were performed, and the protein-protein interaction (PPI) network was constructed with the String website. The prognostic analysis was carried out with multivariate Cox regressions models.

**Results:**

15,818 AS events in 3955 annotated genes were identified across ten solid tumor types. The different DNA methylation patterns between tumor and normal tissues at the corresponding alternative spliced exon boundaries were shown, and 51.3% of CpG sites (CpGs) revealed hypomethylated in tumors. Notably, 607 CpGs were found to be highly correlated with 369 cancer-specific AS events after permutation tests. Among them, the hypomethylated CpGs account for 52.7%, and the number of down-regulated exons was 173. Furthermore, we found 38 AS events in 35 genes could serve as new molecular biomarkers to predict patient survival.

**Conclusions:**

Our study described the relationship between DNA methylation and AS events across ten human solid tumor types and provided new insights into intragenic DNA methylation and exon usage during the AS process.

## Background

Alternative splicing (AS) is one of the conserved biological processes diversifying the transcriptome and proteome [1]. Pre-mRNA splicing is a key step of gene expression in which introns within nascent RNA are removed, and exons are ligated to form mature mRNA [2]. It is estimated that more than 90% of human genes with multiple exons undergo AS during pre-mRNA maturation, highlighting the importance of AS in determining gene function [3, 4]. Recently, AS has been implicated as an important signature to understand tumorigenesis, cancer progression, and resistance to therapy [5]. Several lines of evidence have shown that the disruption of AS is frequently associated with the inactivation of tumor suppressors and activation of oncogenes [6, 7]. For example, Bcl-x, an apoptosis regulator, is found to be switched from its pro-apoptotic into anti-apoptotic splicing isoforms in a number of cancer types [8, 9]. Another well-characterized example is *CD44*, whose different splicing isoforms have been associated with tumor evasion and metastasis [10, 11]. Moreover, Kahles and colleagues have recently provided a comprehensive landscape of AS events across different tumor types [12]. Other studies have also reported the prognostic value of AS events in multiple cancer types, such as ovarian cancer [13], breast cancer [14], glioblastma [15] and gastrointestinal adenocarcinomas [16], suggesting a predominant role of splicing dysregulation in cancers.

The splicing reaction is generally regulated by *cis*-elements within the pre-mRNA and *trans*-acting splicing factors that bind to these *cis*-elements [17]. Alterations of splicing factors, mutations in spliceosomal proteins, and regulation exerted by pre-mRNA *cis*-elements can all contribute to the splicing alterations in cancers [18–20]. Additionally, epigenetic modification has been recently proposed as another regulator of alternative pre-mRNA splicing patterns [11, 21, 22]. The genome-wide mapping analysis has unveiled that DNA methylation was a strong marker for exon boundaries and suggested the possible role of DNA methylation in exon definition and splicing regulation [23, 24]. Anastasiadou et al. have reported that the increased CpG methylation was frequent in alternatively spliced sites [25]. In support, by studying wild-type and methylation deficient embryonic stem cells, Yearim et al. have found that DNA methylation could affect the splicing of more than 20% of alternative exons [26].

The global change of splicing in tumor tissues in comparison to their normal tissue counterparts is increasingly appreciated. However, the regulatory mechanisms underlying cancer-specific AS events, especially the influence of DNA methylation on AS, remain poorly understood. To figure out the association between AS events and DNA methylation of alternatively spliced exons, we first compared the transcriptome-wide splicing between tumor and matched normal tissues across ten solid tumor types. Further, we compared the DNA methylation of CpG sites (CpGs) at the boundaries of alternatively spliced exons. Finally, we utilized correlation analysis and permutation test to assess whether there is an association between DNA methylation and cancer-specific AS events. By these means, we expect to explore the role of methylation in exon usage during transcriptional process and carcinogenesis.

## Methods

### Overall workflow

**Fig. 1:**
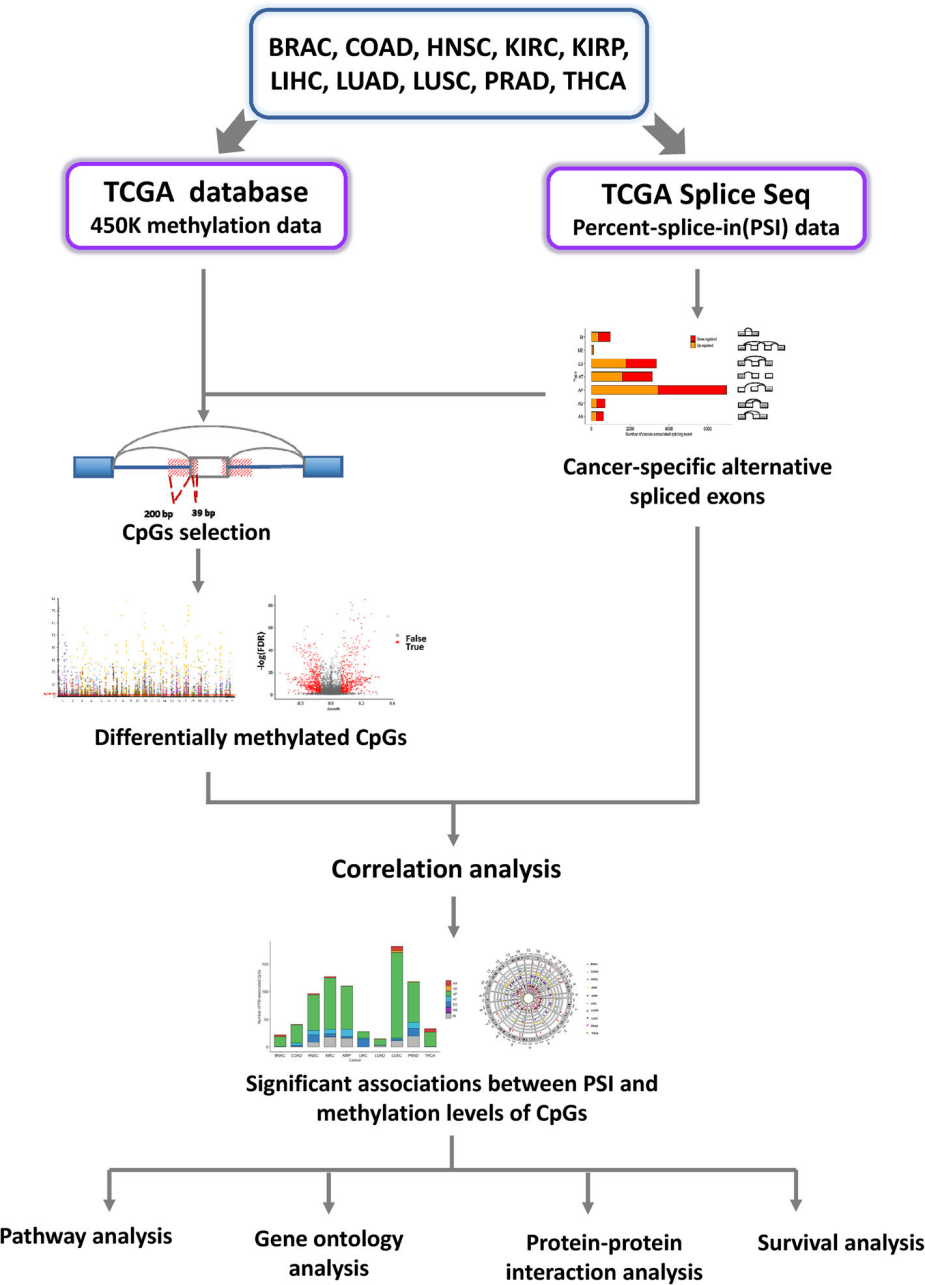
Flowchart of the present study

The overall workflow of the present study is shown in Fig. 1. Using TCGA SpliceSeq dataset for ten solid tumor types, AS events were analyzed in 580 paired tumor-normal tissues. Then, the methylation levels of CpGs at the boundaries of alternatively spliced exons were compared between 641 normal and tumor samples from TCGA. Subsequently, we correlated cancer-specific AS events with methylation features and observed a significant association between them. To further understand the function and mechanism of the genes containing methylation-associated cancer-specific AS events, functional enrichment, and network analyses were applied. Finally, we found that a set of cancer-specific AS events could serve as reliable prognostic biomarkers for cancers.

### Data acquisition

Data on transcriptome alterations were downloaded from TCGA SpliceSeq portal (http://bioinformatics.mdanderson.org/TCGASpliceSeq/). TCGA SpliceSeq is a web-based bioinformatics resource containing the mRNA splicing patterns of 33 types of TCGA cancers and adjacent normal samples. SpliceSeq can be used to calculate the “percent spliced in” (PSI) value for each splicing event in each cancer sample [3], which provides a clear view of the splice junction and the proportion of exons included in different samples. PSI values were utilized to quantify seven types of AS events: exon skip (ES), alternate donor site (AD), alternate acceptor site (AA), retained intron (RI), mutually exclusive exons (ME), alternate terminator (AT) and alternate promoter (AP) [27]. In the present study, AS events (379,749 in total) that have PSI values in more than 75% of samples were included.

Data of somatic mutations, copy number variations, mRNA expression, and DNA methylations were downloaded from publicly released TCGA level 3 data (processed data from USCS Cancer Genome Browser). In this study, ten types of cancers were included: breast invasive carcinoma (BRCA), colon adenocarcinoma (COAD), head and neck squamous cell carcinoma (HNSC), kidney renal clear cell carcinoma (KIRC), kidney renal papillary carcinoma (KIRP), liver hepatocellular carcinoma (LIHC), lung adenocarcinoma (LUAD), lung squamous cell carcinoma (LUSC), prostate adenocarcinoma (PRAD) and thyroid carcinoma (THCA). Illumina Human Methylation 450 BeadChip (450 K array) was used for DNA methylation data. Patient clinical information was also downloaded from TCGA dataset. The sample size of each cancer is summarized in Additional file 1: Table S1.

Gene coordinates and RefSeq annotations were obtained from UCSC (Jul 2013 release, hg 19) [28]. All the features of exons and introns referred to RefSeq genes. The ‘exon boundary’ was determined as 200 nucleotides in size for intronic regions and 39 nucleotides for exonic regions [29].

### Identification of cancer-specific AS events and methylated CpGs

Wilcoxon paired test was applied to compare the PSI values between tumor tissues and matched adjacent normal tissues. In each cancer type, we identified cancer-specific AS events that satisfied the following criteria: (1) the distributions of PSI values were significantly different between tumor and normal tissues (FDR < 0.05); (2) the change of PSI values (**|**△PSI**|**) between tumor and normal tissues was greater than 0.1. Differentially methylated CpGs were detected by applying a paired *t*-test, setting the absolute average methylation change (**|**△meth**|**) ≥ 0.1 and FDR < 0.05 as selection criteria. All statistical analyses were performed using R software (version 3.6.0).

### Correlation analysis and permutation test

Patients with both splicing information and methylation data were used for correlation analysis. Covariance analysis model, after adjusting for copy number variants and somatic mutation in tumor, as well as Spearman correlation analysis in normal tissues, were performed to investigate the correlations between PSI and DNA methylation respectively. In order to infer their statistical significance, we further carried out the permutation test. The number of permutations was 1000, and we considered the permutation *P* < 0.05 was statistically significant. In the present study, the aovperm() function in R package “permuco” and spearman_test() function in package “coin” were used for permutation tests.

### Functional enrichment analysis

Gene Ontology (GO) enrichment and Kyoto Encyclopedia of Genes and Genomes (KEGG) pathway analyses were conducted on genes with methylation-associated cancer-specific AS events using “ClusterProfiler” package in R software [30]. GO and KEGG enrichment analyses were based on the threshold of FDR < 0.05.

### Protein-protein interaction analysis

Genes with significantly methylation-associated AS events were analyzed through the protein-protein interaction (PPI) network. The PPI analysis was constructed with the Search Tool for the Retrieval of Interacting Genes/Proteins (STRING, https://string-db.org/cgi/input.pl) [31]. The confidence score ≥ 0.4 and the maximum number of interactors = 0 were set as the selection criteria.

### Survival analysis

To assess the prognostic value of cancer-specific AS events and methylated CpGs, we performed survival analysis by using the “survival” package in R. The Cox proportional hazard model was utilized to test the interactions between different variables and overall survival in multivariate analysis by adjusting for age, gender, TNM stage and adjuvant therapy (including chemotherapy and radiotherapy). *P* < 0.05 was considered as the threshold for significance.

## Results

### Identification of cancer-specific AS events in common solid tumors

**Fig. 2:**
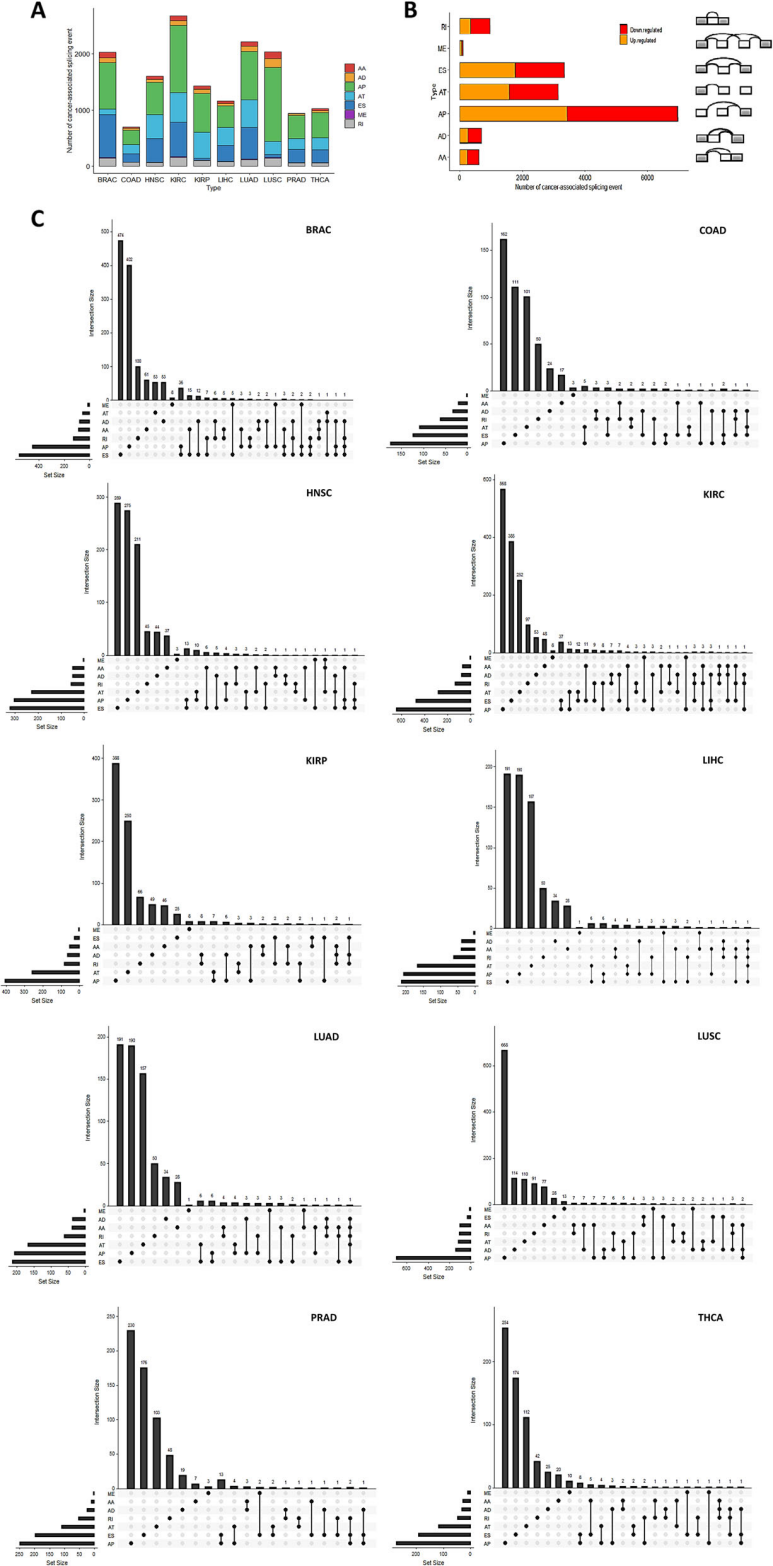
Cancer-specific AS events across human solid tumors. **a** Bar plot showing the numbers of cancer-specific AS events in different tumors split according to types of event. **b** Bar plot showing the numbers of cancer-specific AS events in seven different types of AS. **c** The UpSet plot of interactions between the seven types of cancer-specific AS events. One gene may have several types of AS events

To systematically characterize abnormal AS events in common solid tumors, we compared PSI values between tumors and matched adjacent normal tissues. We found that 15,818 AS events in 3955 annotated genes were significantly altered in cancer vs. normal tissues. Splicing alterations were most abundant in KIRC and LUAD, while least in COAD (Additional file 1: Table S2). For KIRC, a total of 2678 differential AS events in 1550 genes were identified, including 81 AAs in 74 genes, 91 ADs in 83 genes, 1190 APs in 643 genes, 535 ATs in 286 genes, 611 ESs in 474 genes, 12 MEs 12 genes, and 142 RIs in 137 genes. For LUAD, a total of 2212 differential AS events in 1369 genes were identified (Fig. 2a).

For the seven AS events types, we found that AP and ES took up 44.0 and 21.2% of the AS events, respectively, followed by AT (19.9%). In addition, we observed that the numbers of AS events with increased vs. decreased PSI values were similar, with the exception of RI and AD events. (Fig. 2b).

Notably, one gene could possess more than one cancer-specific AS events. The numbers of genes that possess differential AS events in varied cancer types are depicted in Fig. 2c.

### Identification of differentially methylated CpGs at exon boundaries

**Fig. 3:**
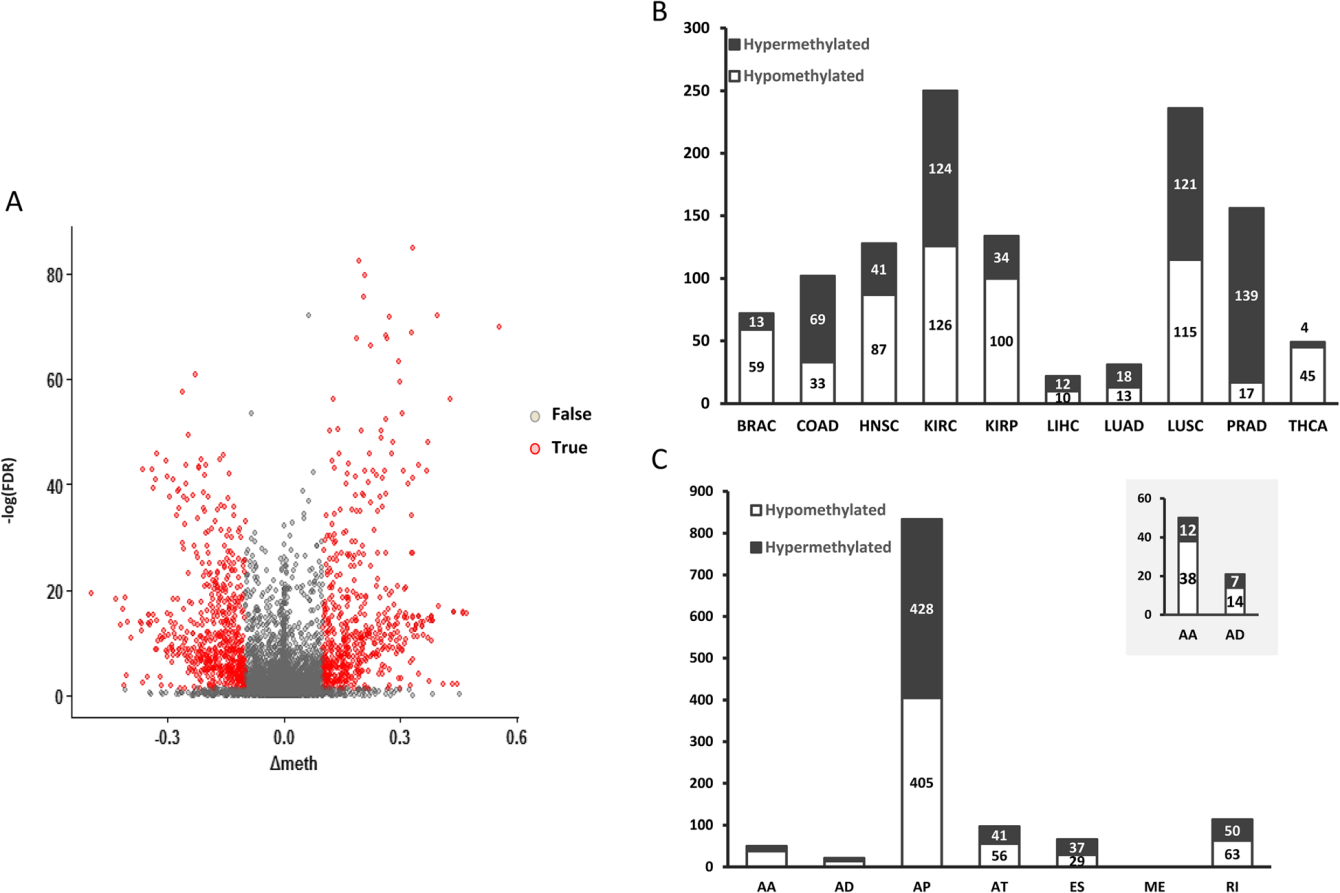
Differentially methylated CpGs at alternatively spliced exon boundaries in multiple tumors. **a** Volcano plot showing a comparison of the methylation level of CpGs for tumor tissues versus adjacent normal tissues. This plot depicts the biological significance (|△meth|) ≥ 10%) on the X axis and the statistical significance (−log10 FDR) on the Y axis. **b** The proportion of hypomethylated and hypermethylated CpGs at cancer-specific alternative spliced exon boundaries in different tumors. **c** The proportion of hypomethylated and hypermethylated CpGs at cancer-specific alternative spliced exon boundaries in different splicing types

To examine the differences of DNA methylation, we retrieved methylation data of CpGs at exon boundaries from TCGA data portal. After comparing between tumor and matched normal tissues, we identified a large amount of CpGs with differential methylation levels (Fig. 3a). Totally, 1180 CpGs were detected, among which KIRC displayed the highest number of CpGs, including 5 CpGs in AA, 3 CpGs in AD, 186 CpGs in AP, 23 CpGs in AT, 15 CpGs in ES and 18 CpGs in RI (Additional file 1: Table S3). We also observed that the proportions of hypomethylated CpGs in different solid tumors were similar, with the exception of COAD (32.4%) and PRAD (10.9%) (Fig. 3b). When comparing the differentially methylated CpGs in varied types of AS events, 833 CpGs were identified in AP type, of which 48.6% were hypomethylated. The proportions of hypomethylated CpGs in other types of AS were similar, ranging from 43.9% in ES to 66.7% in AD **(**Fig. 3c). Taken together, hypomethylation was a more frequently observed form of differential methylation in tumor tissues, compared to hypermethylation.

### Correlation between cancer-specific AS events and DNA methylation

**Fig. 4:**
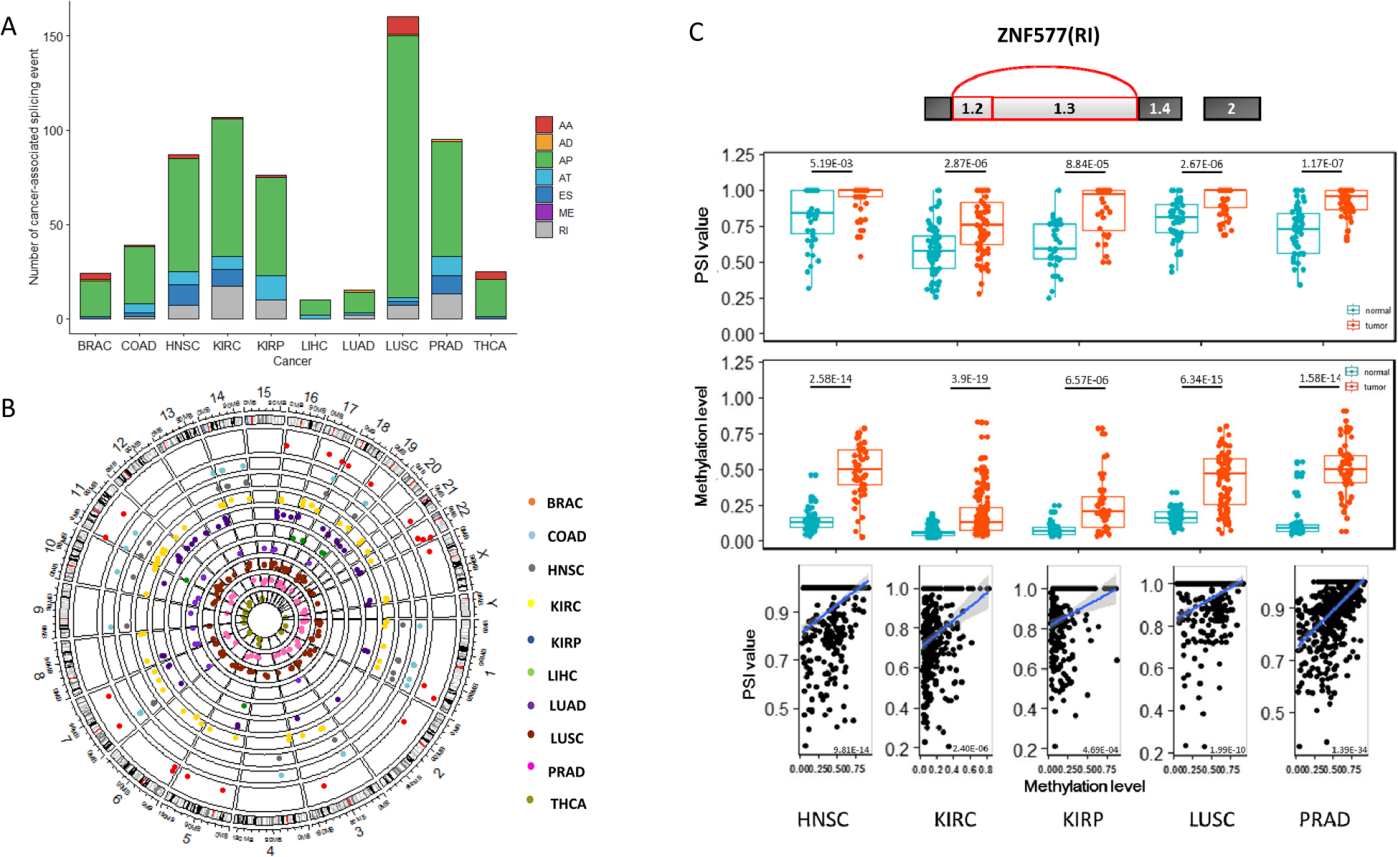
A significant correlation between cancer-specific AS events and DNA methylation at exon boundaries. **a** Column plot of the number of alternative splicing-related CpGs in different cancer types split according to type pf event. **b** Circos plot showing the distribution of methylation-associated splicing events across the human genome in ten solid tumors. The track outside the chromosome track showed the distribution of gene density across the genome. Inside tracks denote the Spearman correlation coefficient (ranging from − 1 to 1) between PSI and methylation level across the genome in each human tumor (from outside to inside): BRAC, COAD, HNSC, KIRC, KIRP, LIHC, LUAD, LUSC, PRAD, THCA. **c** Common change of *ZNF577* in five cancer types. Upper: Box plot of PSI values of exon 1.2 and 1.3 in *ZNF577* in tumor and normal samples in five cancer types. Middle: Box plot of the methylation level of CpG site (cg11269599) in *ZNF577* in tumor and normal samples in five cancer types. Lower: Scatter plot showing the significant relationship between PSI values and methylation levels in five cancer types

To explore possible regulatory mechanisms of cancer-specific AS, we further applied correlation analysis between the PSI values and methylation levels of CpGs at alternatively spliced exon boundaries. A significant association between PSI and DNA methylation was observed from the covariance analysis model after adjusting for covariates, including copy number variants and somatic mutation. After performing the permutation test, 891 CpGs were found to be highly correlated with 483 cancer-specific AS events (permuted *P* < 0.05) (Additional file 1: Table S4). To better determine whether the changes in AS resulted from changed methylation levels or vice versa, we also performed association analysis in the normal tissues. Results showed that the methylation levels of 607 CpGs were not significantly correlated to PSI in the normal tissues after permutation tests (permuted *P* < 0.05) (Additional file 1: Table S4). The number of PSI-associated CpGs is presented in Fig. 4a. Among them, 320 CpGs were hypomethylated, and 287 were hypermethylated in tumor tissues. Expect for COAD, LIHC, LUAD, LUSC, and PRAD, the hypomethylated CpGs account for more than half of AS-associated CpGs in cancers (ranging from 54.5 to 95.7%)(Additional file 2: Figure S1-A). Among the exons, 173 exons were down-regulated (ranging from 35.7% in THCA to57.6% in LUSC) in the tumor tissues (Additional file 2: Figure S1-B).

Though the regions of splicing-related CpGs showed differently across varied chromosomes and regions (Fig. 4b), some common features in different tumor types were sill discovered. The common AS events having similar relationships with CpGs, which changed in at least three cancer types are shown in Table 1. This result suggested that there were indeed a significant number of AS-associated CpGs shared by multiple cancer types. Taking *ZNF577* and cg11269599 as examples, we found that the exon 1.2 and exon 1.3 of *ZNF577* showed increased PSI values in five cancer types when comparing tumors to the cognate normal tissues. The CpG site, cg11269599, also showed significantly hypermethylated in HNSC, KIRC, KIRP, LUSC, and PRAD. The correlations between both aforementioned were also observed consistently in the five cancer types (Fig. 4c). The preference of a specific splice variant by several cancer types could help to confirm the link between AS and DNA methylation [32].

**Table 1:** Splicing events and DNA methylation altered in the same direction in at least three cancer types

Gene name	Tumor types altered significantly	CpG site	Exon	Type of AS	The direction of the correlation
ZNF577	HNSC,KIRC,KIRP,LUSC,PRAD	cg11269599, cg10635122, cg03562414	1.2:1.3	RI	+
	KIRC,KIRP,PRAD	cg10783469, cg24794228, cg23010048, cg16731240	1.2:1.3	RI	+
EGFLAM	BRAC,HNSC,KIRC,THCA	cg21201393	1.2:1.3	RI	+
ANK3	COAD,LUAD,LUSC	cg22601415	20.1	AP	–
DNASE1L1	KIRC,KIRP,THCA	cg21459545	28	AP	–
LTB4R2	KIRC,LUSC,PRAD	cg07164388	3.1	AP	–
PIK3R1	HNSC,LUSC,THCA	cg25091228	1.2:1.3	RI	+
TNFRSF10C	HNSC,KIRC,KIRP,PRAD	cg14015044	9	AP	–

*Abbreviations*: *BRAC* Breast invasive carcinoma, *HNSC* Head, and neck squamous cell carcinoma, *KIRC* Kidney renal clear cell carcinoma, *KIRP* Kidney renal papillary carcinoma, *LUAD* Lung adenocarcinoma, *LUSC* Lung squamous cell carcinoma, *PRAD* Prostate adenocarcinoma, *THCA* Thyroid carcinoma, *AS* Alternative splicing, *AP* Alternate promoter, *RI* Retained intron, *AT* Alternate terminator

“+” represents the positive correlation between PSI values and DNA methylation in the tumor tissues. “-” represents the inverse correlation between PSI values and DNA methylation in the tumor tissues

### Gene functional enrichment analysis and network construction

We further conducted GO analysis on genes containing methylation-related cancer-specific AS events for different cancer types. Results showed that these genes were enriched in different GO terms across different cancers. In COAD, high enrichment of genes was associated with cellular components, including sarcolemma and basal part. In THCA, genes were mainly associated with molecular functions, including hormone binding and acyl-CoA ligase activity (Additional file 1: Table S5). KEGG pathway analysis was also performed. Only the pathway “Glycerophospholipid metabolism” was found significantly enriched by genes in LIHC (Additional file 1: Table S6). These results suggested that the activity of these genes might be in an independent manner in different cancer types.

Moreover, we constructed a PPI network using the STRING database. In the PPI network, *NCK1* and *DOK1* were the top hub genes in the HNSC network. *PIK3R1 PRKACA* and *LCK* were the top three hub genes in the LUSC network. However, in other cancers, no PPI networks were constructed with statistical significance (Additional file 3: Figure S2).

### Survival analysis of AS events and CpGs

We then explored whether the associated AS events and CpGs could be effectively used as a prognostic signature for each cancer. Cox regression analysis was performed adjusting for age, gender, TNM stage and adjuvant therapy, and results showed that 10.3% (38/369) AS events could significantly distinguish patients with longer versus shorter survival prognoses (Table 2). Because splicing of introns could be regulated under a co-transcriptional mechanism [33], an alteration in gene expression may affect AS of corresponding genes. Thus, we examined mRNA expression of genes containing cancer-specific AS events and found that the majority of them (85.71%, 30/35) were not significantly associated with survival, suggesting that these AS events are partially influenced by their gene expression (Table 2). Additionally, we evaluated DNA methylation of CpGs at the survival-related AS exons boundaries. 14 (20.6%) CpGs in 8 (21.1%) AS exons were found significantly associated with the overall survival of cancer patients. (Table 2).

**Table 2:** Multivariate Cox proportional hazard model analyses of associations between alternative exons, methylation of corresponding CpG and cancer patients’ survival

Tumor type	Gene expression			PSI			Methylation level		
	Gene	*P*	HR(95%CI)	Alternative Exon	*P*	HR(95%CI)	CpGs	*P*	HR(95%CI)
BRAC	FAXDC2	0.558	0.96 (0.84,1.10)	3	0.036	3.98 (1.09,0.04)	cg18260397	0.428	0.57 (0.14,2.31)
COAD	OBSCN	0.753	0.97 (0.78,1.20)	118	0.001	0.20 (0.20,0.08)	cg25318301	0.380	0.48 (0.09,2.49)
							cg03669282	0.581	0.72 (0.23,2.31)
HNSC	ABL2	0.489	1.10 (0.84,1.42)	2	0.020	4.59 (1.27,16.58)	cg03471346	0.095	0.40 (0.14,1.17)
HNSC	DNASE1L1	0.003	1.45 (1.13,1.86)	3.1	0.007	4.40 (1.49,12.95)	cg21459545	0.788	0.84 (0.25,2.90)
HNSC	RARA	0.393	1.16 (0.82,1.63)	5	0.009	0.08 (0.01,0.54)	cg05824218	0.350	1.93 (0.49,7.64)
KIRC	ATG16L2	0.104	1.14 (0.97,0.10)	4.1:4.2	0.011	6.79 (1.55,29.83)	cg21806242	0.187	13.92 (0.28,698.07)
KIRC	EGFLAM	0.957	1.00 (0.85,1.17)	20.1	0.047	0.43 (0.18,0.99)	cg21201393	0.197	4.73 (0.45,50.22)
KIRC	EVL	0.004	1.47 (1.13,1.90)	3	0.027	0.32 (0.12,0.88)	cg18621299	0.885	0.84 (0.07,9.47)
KIRC	FMNL1	0.318	1.13 (0.89,1.42)	13.1	0.010	3.59 (1.37,9.44)	cg19735250	0.261	2.95 (0.45,19.42)
KIRC	NAV2	0.276	1.17 (0.88,1.54)	17	0.014	96.94 (2.48,3787.07)	cg19222784	0.857	0.82 (0.10,6.76)
							cg20949700	0.997	1.00 (0.13,7.54)
KIRC	SIGIRR	0.668	1.06 (0.80,1.40)	2	0.041	6.29 (1.08,36.63)	cg02585906	0.173	0.24 (0.03,1.88)
							cg23029021	0.005	0.08 (0.01,0.47)
KIRC	TBC1D14	0.908	0.98 (0.65,1.46)	4	0.024	26.17 (1.52,449.63)	cg16851482	0.778	1.44 (0.12,17.83)
KIRC	ZNF577	0.975	1.00 (0.82,1.22)	1.2:1.3	0.009	6.05 (1.57,23.33)	cg03562414	0.471	1.59 (0.45,5.62)
							cg10635122	0.320	2.03 (0.50,8.22)
							cg11269599	0.007	7.39 (1.74,31.47)
							cg10783469	0.214	2.73 (0.56,13.32)
							cg16731240	0.030	4.41 (1.16,16.78)
							cg23010048	0.053	4.04 (0.98,16.61)
							cg24794228	0.228	2.28 (0.60,8.69)
KIRP	C19orf25	0.012	0.50 (0.29,0.86)	2.4	0.006	0.01 (0.00,0.26)	cg16613938	0.665	0.55 (0.04,8.14)
							cg23291200	0.980	0.94 (0.01,163.12)
KIRP	CABP1	0.0004	0.72 (0.60,0.87)	3.1	0.004	59.40 (3.63,972.14)	cg24292016	0.011	0.02 (0.00,0.42)
							cg25969992	0.011	0.04 (0.00,0.49)
KIRP	CALD1	0.632	0.88 (0.52,1.49)	2	0.013	8.85 (1.58,49.62)	cg04874031	0.007	149.57 (3.97,5636.65)
							cg16253634	0.019	148.62 (2.24,9849.90)
							cg24956866	0.027	97.96 (1.70,5637.50)
KIRP	CALD1	0.632	0.88 (0.52,1.49)	5	0.013	0.11 (0.02,0.63)	cg04874031	0.007	149.57 (3.97,5636.65)
							cg16253634	0.019	148.62 (2.24,9849.90)
							cg24956866	0.027	97.96 (1.70,5637.50)
KIRP	CCL28	0.918	1.01 (0.82,1.25)	2	0.009	7.17 (1.64,31.37)	cg04187185	0.365	0.50 (0.11,2.26)
							cg16324633	0.027	10.72 (1.31,87.54)
KIRP	CIRBP	0.004	0.38 (0.20,0.73)	8.2:8.3	0.011	82.29 (2.75,2460.57)	cg02644867	0.480	3.51 (0.11,113.99)
KIRP	DNASE1L1	0.118	2.15 (0.82,5.63)	3.1	0.030	29.90 (1.40,637.29)	cg21459545	0.251	0.32 (0.05,2.24)
							cg22562219	0.081	0.08 (0.01,1.36)
							cg24834461	0.224	0.20 (0.02,2.63)
KIRP	PIGQ	0.703	0.88 (0.46,1.68)	3	0.047	0.05 (0.00,0.96)	cg03815552	0.575	0.23 (0.00,38.74)
KIRP	SLC9A3R2	0.650	1.11 (0.70,1.78)	3	0.020	0.05 (0.00,0.62)	cg08601673	0.263	0.16 (0.01,3.97)
KIRP	TM4SF18	0.001	0.58 (0.41,0.81)	2.1	0.015	0.04 (0.00,0.55)	cg11011913	0.087	4.98 (0.79,31.30)
LIHC	C1QTNF1	0.580	1.03 (0.93,1.13)	5	0.008	0.27 (0.10,0.70)	cg23882796	0.553	1.24 (0.61,2.51)
LUAD	GALK2	0.013	1.58 (1.10,2.26)	3.1	0.002	13.29 (2.53,69.98)	cg08036668	0.219	0.42 (0.11,1.67)
LUSC	AHCYL2	0.158	1.14 (0.95,1.36)	3	0.031	1.88 (1.06,3.33)	cg15664152	0.933	1.03 (0.51,2.06)
LUSC	AQP1	0.022	1.13 (1.02,1.26)	7.1	0.046	3.51 (1.02,12.06)	cg07135629	0.952	1.04 (0.32,3.41)
							cg04551925	0.790	0.86 (0.28,2.65)
							cg10132917	0.645	1.29 (0.44,3.82)
							cg11827925	0.141	1.95 (0.80,4.73)
							cg04372674	0.049	2.22 (1.00,4.89)
							cg25075794	0.047	2.20 (1.01,4.79)
							cg25230363	0.038	2.42 (1.05,5.57)
LUSC	AQP1	0.022	1.13 (1.02,1.26)	7.1	0.046	3.51 (1.02,12.06)	cg26923410	0.052	2.42 (0.99,5.90)
							cg15373767	0.122	2.07 (0.82,5.21)
							cg18080604	0.049	2.74 (1.00,7.50)
							cg09676669	0.078	2.31 (0.91,5.84)
LUSC	ARHGAP24	0.279	0.92 (0.80,1.07)	6	0.042	145.84 (1.20,17,748.46)	cg14376467	0.908	1.04 (0.52,2.09)
							cg13889934	0.997	1.00 (0.48,2.11)
							cg21446955	0.892	1.05 (0.49,2.24)
LUSC	ERG	0.738	1.03 (0.86,1.24)	5	0.038	2.25 (1.05,4.86)	cg01613817	0.581	0.78 (0.32,1.91)
LUSC	FAXDC2	0.285	1.07 (0.94,1.22)	5.1	0.047	2.09 (1.01,4.31)	cg02379533	0.624	0.76 (0.25,2.28)
LUSC	GCNT2	0.970	1.00 (0.89,1.12)	6.1	0.012	2.21 (1.19,4.11)	cg13411789	0.146	0.66 (0.37,1.16)
LUSC	IL1RN	0.721	0.98 (0.89,1.08)	3	0.046	0.55 (0.31,0.99)	cg11783497	0.833	1.11 (0.42,2.89)
LUSC	NAV1	0.569	0.96 (0.85,1.09)	6	0.024	0.49 (0.26,0.91)	cg01411786	0.765	1.16 (0.44,3.03)
							cg05091570	0.982	0.99 (0.39,2.50)
							cg13877974	0.356	0.55 (0.15,1.97)
							cg16023122	0.334	0.48 (0.11,2.13)
							cg01828733	0.350	0.59 (0.19,1.79)
LUSC	PLEC	0.133	1.13 (0.96,1.32)	4	0.006	23.29 (2.50,216.60)	cg00329447	0.117	0.53 (0.24,1.17)
							cg06452769	0.033	0.41 (0.18,0.93)
PRAD	EXOC7	0.346	0.61 (0.22,1.70)	8.2	0.038	40.29 (1.22,1329.36)	cg04470878	0.660	4.86 (0.00,5523.54)
PRAD	FHAD1	0.358	1.14 (0.86,1.53)	15.2	0.041	0.26 (0.07,0.95)	cg07312051	0.675	5.04 (0.00,9748.17)
PRAD	PCDHA9	0.592	0.94 (0.75,1.17)	1.2	0.007	14.70 (2.12,102.01)	cg18029321	0.548	28.37 (0.00,1,537,765.42)
THCA	EGFLAM	0.828	1.06 (0.65,1.73)	20.1	0.028	0.10 (0.01,0.78)	cg21201393	0.705	1.97 (0.60,64.65)

*Abbreviation*: *KIRC* Kidney renal clear cell carcinoma, *KIRP* Kidney renal papillary carcinoma, *LUSC* Lung squamous cell carcinoma, *HR* Hazard ratio

The Cox regression model adjusted for age, gender, TNM stage, and adjuvant therapy

## Discussion

Collectively, we performed a comprehensive analysis of the relationship between cancer-specific AS events and DNA methylation. We found that approximately half of the AS events were AP and ES types in human solid tumors. The boundaries of alternatively spliced exons were more likely to be hypomethylated in tumor tissues compared with adjacent normal tissues. Association analyses and permutation tests revealed that cancer-specific AS events were significantly correlated with DNA methylation. Our research provided a novel perspective of the regulatory mechanism of cancer-specific AS.

It is well accepted that AS plays an important role in multiple cellular processes and development programs, as well as contributing to tumorigenesis [34, 35]. Nonetheless, to our knowledge, few studies have investigated the role of DNA methylation as a regulator of the cancer-specific AS. Recently, Laurent et al. have demonstrated that methylation differences were more prominent at the exon-intron boundaries [36]. Other studies also found that DNA methylation links with the inclusion rate of alternative exons [26, 37]. These findings supported the hypothesis that AS could be functional by *cis*-regulation of DNA methylation. With the advantage of high-throughput data, TCGA data portal provides opportunities for the integration analyses of multi-omics data. TCGA Splice Seq, web-based bioinformatics, providing a clear view of the mRNA splicing patterns of 33 tumor types, across a dataset of more than 10,000 TCGA samples. Ryan et al. identified and calculated each potential splicing event across 33 types of cancer to establish TCGA SpliceSeq database, while did not evaluate the potential mechanism and clinical usage of AS events [27]. In the present study, we integrated AS events from SpliceSeq and TCGA data together to comprehensively explore potential regulatory mechanisms of DNA methylation for cancer-specific AS. We performed an association analysis between cancer-specific AS events and CpGs at their exon boundaries. The parameter selected in this study was based on previous reports [38–40], especially Castle’s study, in which they examined the exon neighborhoods in the size of 200 nucleotides for intronic regions and 39 nucleotides for exonic regions to identify splicing *cis*-regulatory elements in sequences [29].

In the present study, to ensure the AS difference resulted from DNA methylation, we excluded CpGs showing evidence of associations with AS in normal tissues. By using such analysis, we identified a number of PSI-related CpGs, whose locations appeared differently across varied chromosomes and regions in different cancers. Moreover, the higher proportion of decreased methylation levels of AS-associated CpGs were observed in the majority of solid cancer types, except for COAD, LIHC, LUAD, LUSC, and PRAD. We speculated that this was due to the heterogeneity among tumor samples. It is well known that the splicing process is predominantly tissue- and cancer-specific [12, 41]. Meanwhile, DNA methylation is also demonstrated to be varied across tumor types [42]. It is worth to note that several cases showing the same pattern of changes were identified across different cancer types. These signatures were potential candidates for actively selected variants, which might drive tumorigenesis. For example, we found the consistent changes of AS event and cg11269599 in gene *ZNF577* (zinc finger protein 577) across several tumor types. Zinc finger proteins are commonly involved in transcriptional regulation of genes, but the mechanism of how DNA methylation affects AS of *ZNF577* has not been clarified and needs to be determined in further studies [43].

Recently, several studies have given insight into the regulation of DNA methylation in AS. It has been suggested that DNA methylation at exonic regions could affect the binding of methyl-sensitive DNA-binding proteins, such as MeCp2, CTCF, and HP1 [37, 44–46]. MeCp2 was found to be enriched in spliced exons, which could facilitate the recruitment of histone deacetylase, thus causing Pol II pause and exon inclusion [46]. In 2011, Shukla et al. found the DNA methylation-mediated splicing of *CD45* pre-mRNA, which inhibited CTCF binding [37]. HP1 could bring several splicing factors to methylated exons [26]. In addition, DNA methylation had also been reported to determine nucleosome positioning and affected the transcriptional elongation rate [47].

We also investigated the prognostic values of the cancer-specific AS events by conducting Cox regression. We found 38 methylation-related cancer-specific AS events could significantly distinguish patients with longer versus shorter survival prognoses. It should be noted that these identified signatures were overrepresented in renal carcinoma. Renal carcinoma has various histological and molecular subtypes and is characterized by poor prognosis and high recurrence. Until now, several molecular biomarkers have been investigated for renal carcinoma, while none of them have been used in clinical practice [48, 49]. Therefore, the identification of novel and effective prognostic biomarkers is important for patients suffering from renal carcinoma. The signatures identified in the present study in renal cancers were not well reported previously and could provide the basis for further study into the pathomechanisms and serve as potential therapeutic targets. Additionally, 14 CpGs at the exon boundaries of 8 survival-related AS also showed significant association with overall survival, even after adjusting for age, gender, TNM stage, and adjuvant therapy. These results led us to speculate that the identified CpGs might be the specific cancer drivers, and serve as prognostic biomarkers for cancers.

By the integrated analyses of multi-dimensional data, our study revealed a deeper understanding of cancer-specific AS events and their relationships with DNA methylation across different solid tumor types. Another strength of our study was the pan-cancer analysis, which could reveal similarities and specificities across different cancer types, as well as enabled the elimination of false-positive and false-negative calls made in several single-tumor-type projects [50]. Finally, we provided novel mechanisms and therapeutic perspectives for either one or several cancer types. The present study has several limitations. First, our study investigated the methylation-splicing features across multiple cancer types instead of the difference within cancer. In addition, we only studied the differentially methylated CpGs between tumor and normal tissues, which could miss the CpGs with hyper- or hypo-methylation in tumors that were associated with either down- or up-regulation of PSI. Finally, our research was based on the public data which lacked several important clinical features, such as a history of drug use, and has not been studied in survival analysis. Therefore, further research is needed to clarify and demonstrate the nature of the regulation of methylation in AS.

## Conclusion

Overall, our analyses identified a significant association between DNA methylation and cancer-specific AS events. The present study contributes to the understanding of the role of methylation in exon usage during transcriptional process and carcinogenesis. Finally, this relatively small set of AS events and CpGs will facilitate the discovery of critical regulators, which are responsible for splicing dysregulation in cancers and thus can be used as new therapeutic biomarkers.

## Supplementary information

**Additional file 1: Table S1.** The number of samples from TCGA SpliceSeq and TCGA we used in the analyses. **Table S2.** The number and distribution of cancer-specific AS events across different cancers. **Table S3.** The number and distribution of AS-associated CpGs across different cancers. **Table S4.** Correlation of cancer-specific AS and DNA methylation in tumor tissues and normal tissues. **Table S5.** GO biological processes that were enriched by genes harboring differentially regulated exon in ten human cancers. **Table S6.** KEGG pathway enrichment of genes with methylation-related cancer-specific AS events across ten human cancer types.

**Additional file 2: Figure S1**. (A) The proportion of hypomethylated and hypermethylated CpGs at cancer-specific AS exon boundaries which showed association with DNA methylation in different tumors. (B) The proportion of up-regulated and down-regulated cancer-specific AS exons in different tumors.

**Additional file 3: Figure S2.** Protein-protein interaction networks of genes with methylation-associated cancer-specific AS events in different cancers without statistical significance.

## Data Availability

TCGA SpliceSeq portal (http://projects.insilico.us.com/TCGASpliceSeq/PSIdownload.jsp) applies alternative splicing data. Information on somatic mutations, copy number variations, and DNA methylations of the human solid tumors in this study are from the UCSC Cancer Genomics Browser (https://xenabrowser.net/datapages/).

## Abbreviations

AA: Alternate acceptor site
AD: Alternate donor site
AP: Alternate promoter
AS: Alternative splicing
AT: Alternate terminator
BRCA: Breast invasive carcinoma
COAD: Colon adenocarcinoma
CpGs: CpG site
ES: Exon skip
FDR: False discovery rate
GO: Gene Ontology
HNSC: Head and neck squamous cell carcinoma
KEGG: Kyoto Encyclopedia of Genes and Genomes
KIRC: Kidney renal clear cell carcinoma
KIRP: Kidney renal papillary carcinoma
LIHC: Liver hepatocellular carcinoma
LUAD: Lung adenocarcinoma
LUSC: Lung squamous cell carcinoma
ME: Mutually exclusive exons
PRAD: Prostate adenocarcinoma
PSI: Percent spliced in
RI: Retained intron
TCGA: The Cancer Genome Atlas
THCA: Thyroid carcinoma
